# Supplementary material for: The causal relationship between the human gut microbiota and pyogenic arthritis: a Mendelian randomization study
Source: Front Cell Infect Microbiol. 2024 Nov 26;14:1452480. doi: 10.3389/fcimb.2024.1452480 (PMC11629706; doi:10.3389/fcimb.2024.1452480)
Supplement: Supplementary file 1 [file DataSheet1.zip › mendelian test/ebi-a-GCST90027490.csv_forest.pdf]

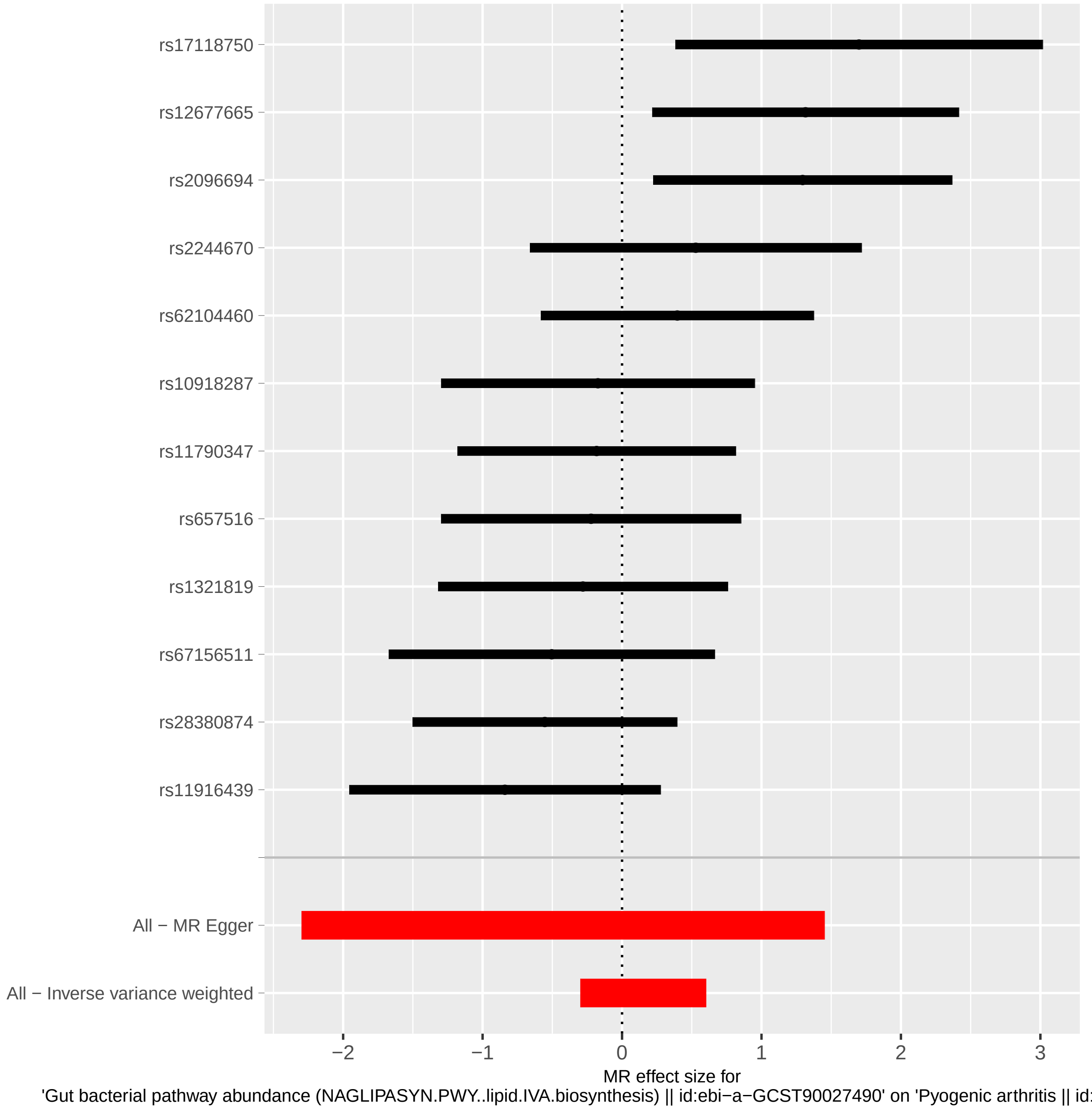
rs17118750
rs12677665
rs2096694
rs2244670
rs62104460
rs10918287
rs11790347
rs657516
rs1321819
rs67156511
rs28380874
rs11916439
All − MR Egger
All − Inverse variance weighted
−2
−1
0
1
2
3
MR effect size for
'Gut bacterial pathway abundance (NAGLIPASYN.PWY..lipid.IVA.biosynthesis) || id:ebi−a−GCST90027490' on 'Pyogenic arthritis || id:
